# Impact of coronavirus disease-2019 on chronic respiratory disease in South Korea: an NHIS COVID-19 database cohort study

**DOI:** 10.1186/s12890-020-01387-1

**Published:** 2021-01-06

**Authors:** Tak Kyu Oh, In-Ae Song

**Affiliations:** Department of Anesthesiology and Pain Medicine, Seoul National University Bundang Hospital, Gumi-ro 173 Beon-gil, Bundang-gu, Seongnam, 13620 Korea

**Keywords:** Asthma, Chronic obstructive pulmonary disease, Interstitial lung diseases, Obstructive sleep apnea

## Abstract

**Background:**

The impact of underlying chronic respiratory diseases (CRDs) on the risk and mortality of patients with coronavirus disease 2019 (COVID-19) remains controversial. We aimed to investigate the effects of CRDs on the risk of COVID-19 and mortality among the population in South Korea.

**Methods:**

The NHIS-COVID-19 database in South Korea was used for data extraction for this population-based cohort study. Chronic obstructive pulmonary disease (COPD), asthma, interstitial lung disease (ILD), lung cancer, lung disease due to external agents, obstructive sleep apnea (OSA), and tuberculosis of the lungs (TB) were considered CRDs. The primary endpoint was a diagnosis of COVID-19 between January 1st and June 4th, 2020; the secondary endpoint was hospital mortality of patients with COVID-19. Multivariable logistic regression modeling was used for statistical analysis.

**Results:**

The final analysis included 122,040 individuals, 7669 (6.3%) were confirmed as COVID-19 until 4 June 2020, and 251 patients with COVID-19 (3.2%) passed away during hospitalization. Among total 122,040 individuals, 36,365 individuals were diagnosed with CRD between 2015 and 2019: COPD (4488, 3.6%), asthma (33,858, 27.2%), ILD (421, 0.3%), lung cancer (769, 0.6%), lung disease due to external agents (437, 0.4%), OSA (550, 0.4%), and TB (608, 0.5%). Among the CRDs, patients either with ILD or OSA had 1.63-fold (odds ratio [OR] 1.63, 95% confidence interval [CI] 1.17–2.26; *P* = 0.004) and 1.65-fold higher (OR 1.65, 95% CI 1.23–2.16; *P* < 0.001) incidence of COVID-19. In addition, among patients with COVID-19, the individuals with COPD and lung disease due to external agents had 1.56-fold (OR 1.56, 95% CI 1.06–2.2; *P* = 0.024) and 3.54-fold (OR 3.54, 95% CI 1.70–7.38; *P* < 0.001) higher risk of hospital mortality.

**Conclusions:**

Patients with OSA and ILD might have an increased risk of COVID-19. In addition, COPD and chronic lung disease due to external agents might be associated with a higher risk of mortality among patients with COVID-19. Our results suggest that prevention and management strategies should be carefully performed.

## Background

After the first report on 31 December 2019, 27 cases of coronavirus disease 2019 (COVID-19) with pneumonia of unknown etiology occurred in Wuhan city, Hubei, China [1]. The COVID-19 has been an outbreak worldwide, and the World Health Organization declared the Chinese outbreak of COVID-19 as a public health emergency of international concern on 30 January 2020 [2] and a pandemic crisis on 11 March 2020 [3]. As of 10 August 2020, approximately 5 million cases of COVID-19, and 150,000 COVID-19 related deaths were reported in the United States [4]. To date, no vaccine is available for COVID-19 [5], and it is currently a public and global health crisis.

Previous studies have identified the important risk factors for worsening outcomes among patients with COVID-19, such as comorbidities, smoking, and obesity [6, 7]. Among the comorbidities, chronic respiratory disease (CRDs) has the third-highest fatality ratio after cardiovascular disease and diabetes [8]. For example, chronic obstructive pulmonary disease (COPD) has been identified as a risk factor for severe status with high mortality in patients with COVID-19 [9]. However, asthma has not been identified as a risk factor for severe outcomes among patients with COVID-19 [10]. Thus, the impact of CRD on outcomes among patients with COVID-19 was reported in previous studies [11, 12]; however, the studies did not focus on the effect of CRD on the risks of COVID-19 among the general population and on patient outcomes. Thus, the relationship between underlying CRD and the risk of COVID-19 among the general population has not been identified. Furthermore, information regarding the risk of mortality in patients with COVID-19 with various CRDs is still lacking.

Therefore, we aimed to investigate various CRDs that affect the risk of COVID-19 among the general population in South Korea. Additionally, we examined the effect of different CRDs on hospital mortality among patients with COVID-19 in South Korea.

## Methods

### Study design and ethical statement

This population-based cohort study was conducted according to the Strengthening the Reporting of Observational Studies in Epidemiology guidelines [13]. The study protocol was approved by the institutional review board of Seoul National University Bundang Hospital (X-2004–604-905) and the Health Insurance Review and Assessment Service (NHIS-2020–1-291). Informed consent was waived because data analyses were performed retrospectively using anonymized data derived from the National Health Insurance Service (NHIS) in South Korea.

### NHIS-COVID-19 cohort database and study population

The NHIS-COVID-19 cohort database was developed for medical research in cooperation between the NHIS and Korea Centers for Disease Control and Prevention (KCDC). The KCDC provides information on patients who were diagnosed with COVID-19 from 1 January 2020 to 4 June 2020, such as the confirmation date of COVID-19, the results of treatment, and the demographic information. However, NHIS and KCDC did not provide the data regarding patients with COVID-19 who were undergoing treatment in the hospital as of June 26th 2020, as their treatment results have not yet been determined. By using this information of patients with COVID-19, the NHIS extracted the control population using stratification methods with regard to age, sex, and residence in February 2020. In the NHIS-COVID-19 cohort database, all disease diagnoses by the International Classification of Diseases (ICD)-10 codes, and prescription information concerning drugs and/or procedures from 2015 to 2020 were included. An independent medical record technician at the NHIS center who was unaffiliated with this study performed data extraction on 26 June 2020. In this NHIS-COVID-19 cohort database, individuals who were ≥ 20 years old were included in the study. In South Korea, patients who were diagnosed with COVID-19 were admitted to the hospital if they had severe symptoms such as pneumonia. However, if they had mild or no symptoms, they were isolated and closely monitored in certain government-managed centers. In addition, the KCDC tested all individuals for COVID-19 in South Korea, who had either direct or indirect contact with COVID-19 patients in the community or hospital.

### CRD

The following diseases were considered as CRDs and the patient data extracted for this study included: COPD (J44*), asthma (J45*), interstitial lung disease (ILD, J84.9), lung cancer (C34*), lung disease due to external agent (J60-J70), obstructive sleep apnea (OSA) (G47.33), and tuberculosis of the lungs (TB, A15). The ICD-10 codes from 2015 to 2019 were used to evaluate CRDs in the study population. The study groups included the CRD group with individuals who were diagnosed with any type of CRD and the control group included the other individuals without any respiratory illnesses. In South Korea, as sole public insurance coverage, the CRDs should be registered in the NHIS database after diagnosis by physicians to receive financial coverage for treatment.

### Endpoints

The primary endpoint of this study was the diagnosis of COVID-19, and it was evaluated from 1 January 2020 to 26 June 2020. The secondary endpoint of this study was hospital mortality among patients who were diagnosed with COVID-19.

### Data collection

Additional data collected included (1) demographic information (age and sex), (2) place of residence (Seoul, Gyeonggi-do, Daegu, Gyeongsangbuk-do, and Other areas), (3) underlying disability, (4) income level in 2020, and (5) the Charlson comorbidity index (CCI), which was calculated based on registered ICD-10 diagnostic codes (Additional File 1) from 1 January 2019 to 31 December 2019. The income level was divided into four groups using quartile ratio, and the patients were divided into seven age groups (20–29, 30–39, 40–49, 50–59, 60–69, 70–79, and ≥ 80 years). The annual income level of all individuals in South Korea is registered to determine yearly NHIS premiums. For this study, income levels were classified into quartile groups. In addition, in South Korea, the total disability of all individuals should be registered in the NHIS database to receive various benefits; it includes physical and brain lesion disabilities; visual disturbance; hearing and speech disabilities; autism; intellectual, mental, renal, heart, and respiratory disorders; hepatopathy; facial disfigurement; intestinal and urinary fistulae; and epilepsy.

### Statistical analysis

The baseline characteristics of all individuals in this study were presented as percentages for categorical variables and mean values with standard deviations for continuous variables. Comparison of characteristics between the CRD and control groups was performed using the t-test for continuous variables and the chi-squared test for categorical variables. We constructed a multivariable logistic regression model to investigate whether CRD was associated with the progression of COVID-19 in South Korea, and it was defined as multivariable model 1. All covariates were included in the model for multivariable adjustment, however, the CCI was included in the other model to avoid multi-collinearity with other underlying diseases that were used for the CCI calculation. In addition, as a sensitivity analysis, we divided the CRD group into seven disease groups and included them in the multivariable logistic regression model for the analysis of the progression of COVID-19 in South Korea, and it was defined as multivariable model 2. The CCI was included in the multivariable model 2 for adjustment, while other underlying diseases that were used for the CCI calculation were not included in the multivariable model 2.

Further, we developed a multivariable logistic model for hospital mortality among patients who were diagnosed with COVID-19 to investigate whether underlying CRD affected hospital mortality, compared to the control group. The two multivariable models (model 1 and model 2) were also constructed to investigate the association of a CRD and seven disease type of CRD with hospital mortality, separately. Hosmer–Lemeshow statistics was used to confirm the goodness of fit of multivariable models as *P* > 0.05, and it was confirmed that there was no multicollinearity in all multivariable models of the entire cohort with a variance inflation factor of < 2.0. The results of the logistic regression models were presented as odds ratios (ORs) with 95% confidence intervals (CIs). A receiver operator characteristic (ROC) analysis was performed to validate the use of logistic regression analysis for this study. R software (version 3.6.3; R Foundation for Statistical Computing, Vienna, Austria) was used for all analyses, and *P* < 0.05 was considered statistically significant.

## Results

### Study population

**Fig. 1:**
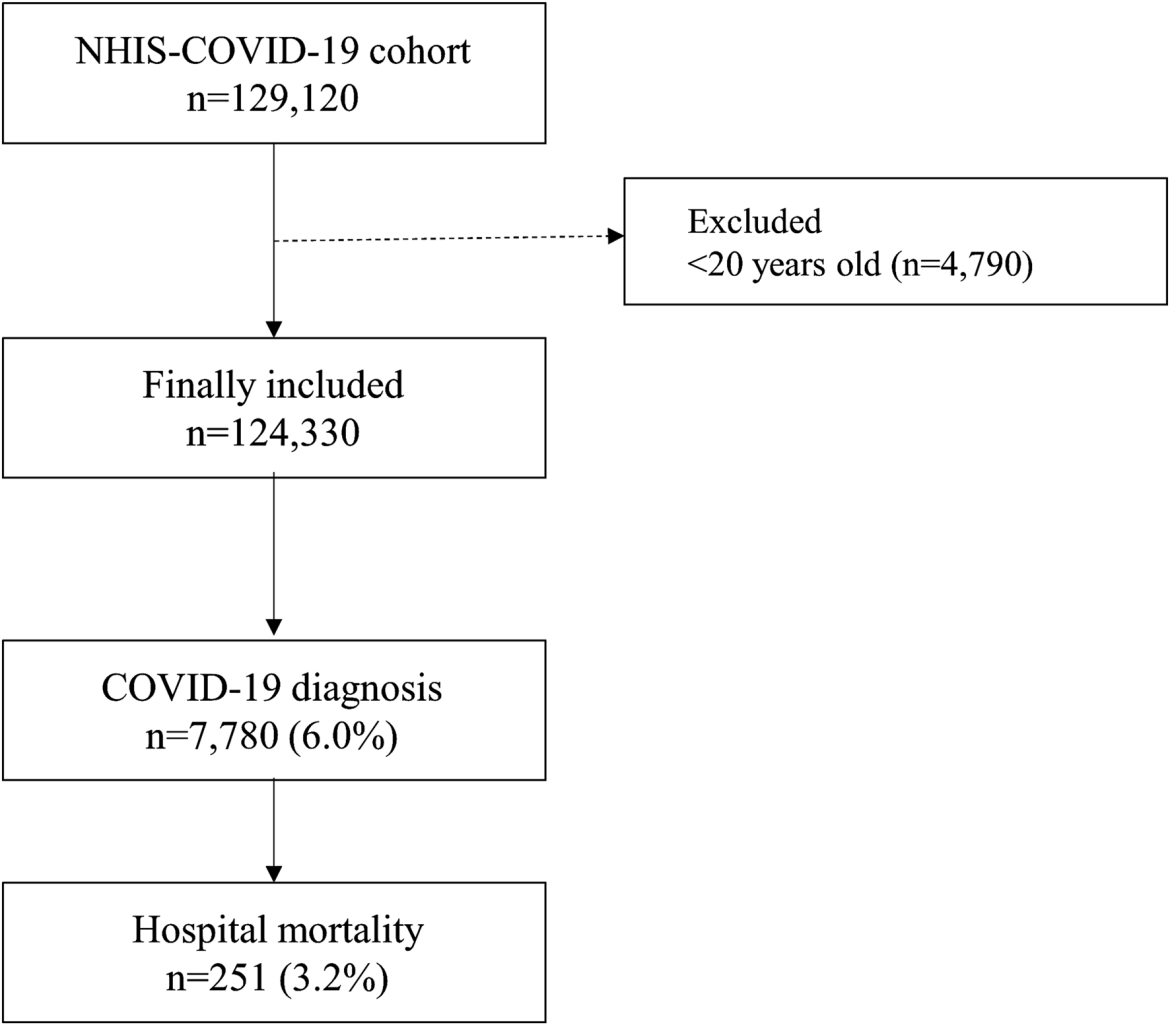
Flow chart depicting selection of individuals for the study

The cohort constituted 129,120 individuals, 4790 of whom were excluded (aged < 20 years). The final analysis included 122,040 individuals; 7669 (6.3%) were confirmed as COVID-19 cases until June 4th 2020. Among the 7669 patients with COVID-19, 251 (3.2%) passed away during hospitalization (Fig. 1). The baseline characteristics of all individuals in the NHIS-COVID-19 cohort are presented in Table 1. There was no missing data in the NHIS-COVID-19 database, except for annual income level in 2121 individuals (1.7%). However, they are not excluded in the analysis, and their income level was included as unknown to avoid bias from excluding them. A total of 36,365 individuals were diagnosed with CRD between 2015 and 2019: COPD (4488, 3.6%), asthma (33,858, 27.2%), ILD (421, 0.3%), lung cancer (769, 0.6%), lung disease due to external agents (437, 0.4%), OSA (550, 0.4%), and TB (608, 0.5%). The results of the comparison of characteristics between the CRD and control groups are presented in Table 2. The CCI in the CRD group was higher than control group (mean value: 2.7 ± 3.3 in CRD group vs 1.5 ± 2.6 in control group; *P* < 0.001).

**Table 1:** Baseline characteristics of all individuals in NHIS-COVID-19 cohort

Variable	Number (%)	Mean (SD)
Age, year		
20–29	32,380 (26.0)	
30–39	13,154 (10.6)	
40–49	16,519 (13.3)	
50–59	25,260 (20.3)	
60–69	19,669 (15.8)	
70–79	10,426 (8.4)	
≥ 80	6922 (9.2)	
Sex, male	48,726 (39.2)	
Residence		
Seoul	8056 (6.5)	
Gyeonggi-do	81,493 (65.5)	
Daegu	6855 (5.5)	
Gyeongsangbuk-do	15,107 (12.2)	
Other area	12,819 (10.3)	
Underlying disability	7655 (6.2)	
Income level		
Q1 (Lowest)	32,387 (26.0)	
Q2	24,979 (20.1)	
Q3	27,774 (22.3)	
Q4 (Highest)	37,069 (29.8)	
Unknown	2121 (1.7)	
Charlson comorbidity index		1.8 (2.9)
Hypertension	32,727 (26.3)	
DM without chronic complication	13,781 (11.1)	
DM with chronic complication	4255 (3.4)	
Peripheral vascular disease	7198 (5.8)	
Renal disease	1392 (1.1)	
Rheumatic disease	2958 (2.4)	
Dementia	3926 (3.2)	
Peptic ulcer disease	9872 (7.9)	
Hemiplegia or paraplegia	568 (0.5)	
Moderate or severe liver disease	146 (0.1)	
Mild liver disease	13,612 (10.9)	
Cerebrovascular disease	5763 (4.6)	
Congestive heart failure	3683 (3.0)	
Myocardial infarction	1187 (1.0)	
Malignancy	22,013 (17.7)	
Metastatic solid tumor	4072 (3.3)	
AIDS/HIV	32 (0.0)	
Any chronic respiratory diseases	36,365 (29.2)	
COPD	4488 (3.6)	
Asthma	33,858 (27.2)	
Interstitial lung disease	421 (0.3)	
Lung cancer	769 (0.6)	
Lung disease d/t external agent	437 (0.4)	
Obstructive sleep apnea	550 (0.4)	
Tuberculosis of lung	608 (0.5)	

SD, standard deviation; DM, diabetes mellitus; AIDS, acquired immune deficiency syndrome; HIV, Human Immunodeficiency Virus

**Table 2:** Comparison of of characteristics between CRD group and control group

Variable	CRD n = 36,365	Control n = 87,965	*P *value
Age, year			< 0.001
20–29	6932 (19.1)	25,448 (28.9)	
30–39	3451 (9.5)	9703 (11.0)	
40–49	4503 (12.4)	12,016 (13.7)	
50–59	6787 (18.7)	18,473 (21.0)	
60–69	6806 (18.7)	12,863 (14.6)	
70–79	4534 (12.5)	5892 (6.7)	
≥ 80	3352 (9.2)	3570 (4.1)	
Sex, male	36,228 (41.2)	12,498 (34.4)	< 0.001
Residence			< 0.001
Seoul	2367 ( 6.5)	5689 ( 6.5)	
Gyeonggi-do	23,442 (64.5)	58,051 (66.0)	
Daegu	2088 ( 5.7)	4767 ( 5.4)	
Gyeongsangbuk-do	4716 (13.0)	10,391 (11.8)	
Other area	3752 (10.3)	9067 (10.3)	
Underlying disability	3109 (8.5)	4546 (5.2)	< 0.001
Income level			< 0.001
Q1 (Lowest)	9758 (26.8)	22,629 (25.7)	
Q2	6681 (18.4)	18,298 (20.8)	
Q3	7815 (21.5)	19,959 (22.7)	
Q4 (Highest)	11,492 (31.6)	25,577 (29.1)	
Unknown	619 (1.7)	1502 ( 1.7)	
Charlson comorbidity index	2.7 (3.3)	1.5 (2.6)	< 0.001
Hypertension	12,896 (35.5)	19,831 (22.5)	< 0.001
DM without chronic complication	5676 (15.6)	8105 ( 9.2)	< 0.001
DM with chronic complication	1897 ( 5.2)	2358 ( 2.7)	< 0.001
Peripheral vascular disease	3306 ( 9.1)	3892 ( 4.4)	< 0.001
Renal disease	663 ( 1.8)	729 ( 0.8)	< 0.001
Rheumatic disease	1325 ( 3.6)	1633 ( 1.9)	< 0.001
Dementia	1940 ( 5.3)	1986 ( 2.3)	< 0.001
Peptic ulcer disease	4397 (12.1)	5475 ( 6.2)	< 0.001
Hemiplegia or paraplegia	255 ( 0.7)	313 ( 0.4)	< 0.001
Moderate or severe liver disease	58 ( 0.2)	88 ( 0.1)	0.007
Mild liver disease	5546 (15.3)	8066 ( 9.2)	< 0.001
Cerebrovascular disease	2635 ( 7.2)	3128 ( 3.6)	< 0.001
Congestive heart failure	1905 ( 5.2)	1778 ( 2.0)	< 0.001
Myocardial infarction	554 ( 1.5)	633 ( 0.7)	< 0.001
Malignancy	8859 (24.4)	13,154 (15.0)	< 0.001
Metastatic solid tumor	1886 ( 5.2)	2186 ( 2.5)	< 0.001
AIDS/HIV	12 ( 0.0)	20 ( 0.0)	0.405

Presented as mean with standard deviation or number with percentage

CRD, chronic respiratory disease; SD, standard deviation; DM, diabetes mellitus; AIDS, acquired immune deficiency syndrome; HIV, Human Immunodeficiency Virus

### Risk of COVID-19 in South Korea

Table 3 shows the results of the multivariable logistic regression model for the progression of COVID-19 in South Korea. In multivariable model 1, the CRD group was not associated with the incidence of COVID-19 compared with the control group (OR 1.04, 95% CI 0.99–1.09; *P* = 0.156). However, in multivariable model 2, the patients with ILD or OSA were associated with 1.63-fold (OR 1.63, 95% CI 1.17–2.26; *P* = 0.004) and 1.65-fold (OR 1.65, 95% CI 1.23–2.16; *P* < 0.001) higher incidence of COVID-19 than the control group. Patients with other CRD, such as asthma *(P* = 0.464), lung cancer (*P* = 0.533), lung disease due to external agents (*P* = 0.061), and TB (*P* = 0.372) were not associated with the incidence of COVID-19. Hosmer–Lemeshow statistics showed that the goodness of fit was appropriate in the models (*P* > 0.05), and the area under curve (AUC) of the multivariable models in ROC analyses was 0.81 (95% CI 0.80–0.81).

**Table 3:** Multivariable logistic regression model for development of COVID-19 in South Korea

Variable	Multivariable model	*P*-value
	OR (95% CI)	
Chronic respiratory diseases group (model1)	1.04 (0.99, 1.09)	0.156
Chronic respiratory diseases: sensitivity analyses (model2)		
COPD	0.96 (0.85, 1.09)	0.553
Asthma	1.01 (0.96, 1.07)	0.464
Interstitial lung disease	1.63 (1.17, 2.26)	0.004
Lung cancer	0.91 (0.67, 1.23)	0.533
Lung disease d/t external agent	1.35 (0.99, 1.85)	0.061
Obstructive sleep apnea	1.65 (1.23, 2.16)	< 0.001
Tuberculosis of lung	0.92 (0.75, 1.11)	0.372
Age, year		
20–29	1	
30–39	0.94 (0.86, 1.02)	0.153
40–49	0.86 (0.80, 0.93)	< 0.001
50–59	0.74 (0.69, 0.79)	< 0.001
60–69	0.60 (0.55, 0.66)	< 0.001
70–79	0.47 (0.42, 0.52)	< 0.001
≥ 80	0.38 (0.33, 0.44)	< 0.001
Income level		
Q1 (Lowest)	1	
Q2	0.80 (0.75, 0.86)	< 0.001
Q3	0.78 (0.73, 0.83)	< 0.001
Q4 (Highest)	0.83 (0.78, 0.88)	< 0.001
Unknown	0.75 (0.62, 0.91)	0.004
Sex, male	1.01 (0.96, 1.06)	0.678
Residence		
Seoul	1	
Gyeonggi-do	0.90 (0.82, 0.99)	0.038
Daegu	0.96 (0.84, 1.10)	0.562
Gyeongsangbuk-do	0.94 (0.84, 1.05)	0.271
Other area	0.89 (0.79, 1.00)	0.053
Underlying disability	1.11 (1.00, 1.22)	0.041
Charlson comorbidity index (model 2)	1.19 (1.18, 1.20)	< 0.0001
Hypertension	0.80 (0.74, 0.85)	< 0.001
DM without chronic complication	1.75 (1.63, 1.88)	< 0.001
DM with chronic complication	0.81 (0.71, 0.92)	0.001
Peripheral vascular disease	0.75 (0.67, 0.83)	< 0.001
Renal disease	0.91 (0.75, 1.11)	0.371
Rheumatic disease	0.98 (0.85, 1.12)	0.734
Dementia	1.96 (1.72, 2.22)	< 0.001
Peptic ulcer disease	1.48 (1.37, 1.59)	< 0.001
Hemiplegia or paraplegia	2.69 (2.11, 3.44)	< 0.001
Moderate or severe liver disease	0.59 (0.33, 1.06)	0.076
Mild liver disease	2.12 (1.99, 2.27)	< 0.001
Cerebrovascular disease	0.99 (0.88, 1.11)	0.886
Congestive heart failure	2.88 (2.60, 3.19)	< 0.001
Myocardial infarction	4.55 (3.95, 5.24)	< 0.001
Malignancy	2.04 (1.93, 2.15)	< 0.001
Metastatic solid tumor	0.95 (0.84, 1.07)	0.387
AIDS/HIV	4.21 (1.84, 9.64)	< 0.001

AUC of the multivariable models: 0.81 (95% CI 0.80 to 0.81)

OR, odds ratio; CI, confidence interval; COPD, chronic obstructive pulmonary disease; DM, diabetes mellitus; AIDS, acquired immune deficiency syndrome; HIV, Human Immunodeficiency Virus

### Hospital mortality among patients with COVID-19

Table 4 shows the results of the multivariable logistic regression model for hospital mortality of COVID-19 patients. In multivariable model 1, the CRD group was not associated with hospital mortality in COVID-19 patients compared with the control group (OR, 1.19; 95% CI, 0.86–1.64; *P* = 0.299). However, in multivariable model 2, the patients with COPD and lung disease due to external agents showed 1.56-fold (OR 1.56, 95% CI 1.06–2.2; *P* = 0.024) and 3.54-fold (OR 3.54, 95% CI 1.70–7.38; *P* < 0.001) higher risk of hospital mortality of COVID-19 patients compared with the control group. Hosmer–Lemeshow statistics showed that the goodness of fit was appropriate in the models (*P* > 0.05), and the AUC of the multivariable models in ROC analyses was 0.83 (95% CI 0.82–0.83).

**Table 4:** Multivariable logistic regression model for hospital mortality in COVID-19 patients (n = 7780, death = 251, 3.2%)

Variable	Multivariable model	*P*-value
	OR (95% CI)	
Chronic respiratory diseases (model 1)	1.19 (0.86, 1.64)	0.299
Chronic respiratory diseases: sensitivity analyses (model 2)		
COPD	1.56 (1.06, 2.2)	0.024
Asthma	1.03 (0.76, 1.41)	0.834
Interstitial lung disease	1.83 (0.74, 4.55)	0.193
Lung cancer	1.82 (0.80, 4.14)	0.154
Lung disease d/t external agent	3.54 (1.70, 7.38)	< 0.001
Obstructive sleep apnea	0.47 (0.06, 3.94)	0.486
Tuberculosis of lung	1.65 (0.48, 5.64)	0.423
Age, 10 year increase	2.85 (2.40, 3.38)	< 0.001
Income level		
Q1 (Lowest)	1	
Q2	0.96 (0.59, 1.57)	0.882
Q3	1.08 (0.70, 1.65)	0.739
Q4 (Highest)	0.89 (0.652, 1.30)	0.554
Unknown	0.67 (0.18, 2.54)	0.560
Sex, male	2.12 (1.55, 2.88)	< 0.001
Residence		
Seoul	1	
Gyeonggi-do	2.26 (0.75, 6.86)	0.148
Daegu	2.60 (0.74, 9.16)	0.136
Gyeongsangbuk-do	2.40 (0.77, 7.49)	0.133
Other area	1.69 (0.50, 5.72)	0.401
Underlying disability	1.33 (0.94, 1.89)	0.109
Charlson comorbidity index, model 2	1.80 (1.32, 2.44)	< 0.001
Hypertension	1.36 (0.89, 2.06)	0.153
DM without chronic complication	1.87 (1.35, 2.59)	< 0.001
DM with chronic complication	1.61 (1.06, 2.45)	0.027
Peripheral vascular disease	1.19 (0.81, 1.76)	0.76
Renal disease	1.47 (0.87, 2.47)	0.148
Rheumatic disease	0.58 (0.30, 1.12)	0.107
Dementia	1.61 (1.11, 2.32)	0.011
Peptic ulcer disease	1.04 (0.73, 1.49)	0.818
Hemiplegia or paraplegia	1.92 (1.03, 3.59)	0.040
Moderate or severe liver disease	5.12 (1.32, 19.90)	0.018
Mild liver disease	0.80 (0.58, 1.10)	0.170
Cerebrovascular disease	0.57 (0.38, 0.87)	0.009
Congestive heart failure	1.91 (1.38, 2.66)	< 0.001
Myocardial infarction	0.79 (0.47, 1.33)	0.374
Malignancy	1.07 (0.78, 1.46)	0.694
Metastatic solid tumor	1.37 (0.85, 2.19)	0.192
AIDS/HIV	1.43 (0.11, 19.37)	0.788

AUC of the multivariable models: 0.83 (95% CI 0.82 to 0.83)

OR, odds ratio; CI, confidence interval; COPD, chronic obstructive pulmonary disease; DM, diabetes mellitus; AIDS, acquired immune deficiency syndrome; HIV, Human Immunodeficiency Virus

## Discussion

In the NHIS-COVID-19 database cohort, individuals with CRDs were not associated with both risk and hospital mortality for COVID-19 in South Korea. However, when the CRDs were divided into seven specific diseases, some CRDs were significantly associated either with a risk of infection or hospital mortality. Specifically, patients with ILD or OSA were associated with a higher incidence of COVID-19, while patients with COPD and lung disease due to an external agent were associated with increased hospital mortality.

OSA was a significant risk factor for COVID-19 among the South Korean population, and this is an important factor in our study. Previous studies reported that obesity was an independent and significant risk factor for poor outcomes in patients with COVID-19 [14–18]. OSA might be one of the main factors that can be used to explain the impact of obesity/OSA on COVID-19 [19]. Obesity is known to be highly correlated with the presence of OSA, and it was undiagnosed in the vast majority [20, 21]. This is because OSA causes decreased lung function, and importantly, increased lung inflammation [22]. In addition, angiotensin-converting enzyme (ACE) plasma activity is known to be increased in untreated patients with OSA [23]. Considering that the increase in ACE2 activity was related to organ injury and infectivity in patients with COVID-19 [24], OSA increases the risk of COVID-19 in patients included in this study via the ACE mechanism. However, our study did not show that OSA was associated with higher mortality of patients with COVID-19; therefore, more studies are needed in this regard.

Our study also reported that ILD patients had a higher risk of COVID-19. Although the exact mechanism is unknown, patients with ILD are known to be more susceptible to viral infection [25] because a respiratory viral infection causes an inflammatory reaction in the lung tissues. With these supporting pieces of evidence, the relationship between viral infection and ILD development is known to be closely linked [26]. Furthermore, patients with ILD often have dyspnea symptoms [27], making it difficult for them to wear masks. Since wearing masks is the most protective method for the prevention of COVID-19 [28], the poor compliance of mask-wearing in patients with ILD might elevate the risk of COVID-19.

Furthermore, we showed that patients with COPD had a 1.56-fold higher risk of hospital mortality after diagnosis of COVID-19. A meta-analysis reported in May 2020 reported that COPD was associated with a higher risk of mortality among patients with COVID-19 [9]. Among the total hospitalized patients with COVID-19, approximately 75% experienced pneumonia and 15% experienced acute respiratory distress syndrome (ARDS) [29], further, COPD is an independent risk factor for higher mortality in patients with pneumonia [30] and ARDS [31]. Thus, COPD can worsen hospital mortality in patients with COVID-19 as reported in a previous study [9]. Furthermore, ACE2, which is known to play an important role in lung injury among patients with COVID-19, was significantly elevated in patients with COPD [32], and they might be at a higher risk of mortality than the control group.

Interestingly, patients with COVID-19 and lung disease due to external agents had the highest risk among CRDs, with an OR of 3.54 (95% CI 1.70–7.38). Patients with COVID-19 and a lung disease due to an external agent might have suffered from pneumoconiosis or pneumonitis due to external agents such as, asbestos, silica, inorganic dust, or chemicals. Although there is a lack of information regarding pneumoconiosis or pneumonitis due to external agents and the prognosis of patients with COVID-19, however, it might be associated with a poor prognosis [33]. Therefore, we can consider that patients with COVID-19 and either pneumoconiosis or pneumonitis due to external agents might suffer from severe pneumonia and ARDS, resulting in higher mortality than the control group.

Our study has several limitations. First, some important variables, including body mass index (BMI), were not included in the analysis because the information was unavailable in the NHIS database. Since obesity is closely related to the development of OSA [34], the lack of information on BMI might affect the results of this study. Second, we did not consider the most important lifestyle factor for CRDs such as smoking history in this study because the NHIS database did not contain the corresponding information. Third, we defined the CRDs and other comorbidities using ICD-10 codes from the NHIS database. However, there is a possibility that some individuals were not diagnosed with comorbidities, including CRDs, because of differences in the accessibility to medical sources. Lastly, a selection bias is possible, as patients with CRD might visit an outpatient clinic or hospital. Therefore, the risk of COVID-19 infection in patients with CRD might differ from other individuals.

## Conclusions

In conclusion, among CRDs, OSA and ILD might increase the risk of COVID-19. In addition, COPD and chronic lung disease due to external agents might be associated with a higher risk of mortality among patients with COVID-19. Our results suggest that prevention and management strategies should be carefully performed.

## Supplementary information

**Additional File 1.** The ICD-10 codes used by comorbidity to compute the Charlson comorbidity index

## Data Availability

The data that support the findings of this study areavailable from National Health Insurance System, but restrictions apply to the availability of these data, which were used under licence for the current study and so are not publicly available. Data are, however, available from the authors upon reasonable request and with permission from the National Health Insurance System (https://nhiss.nhis.or.kr/bd/ab/bdaba000eng.do).

## Abbreviations

ACE: Angiotensin converting enzyme
AUC: Area under curve
CI: Confidence interval
COPD: Chronic obstructive pulmonary disease
COVID-19: Coronavirus disease 2019
CRD: Chronic respiratory disease
ICD-10: International classification of diseases-10 code
ILD: Interstitial lung disease
KCDC: Korea centers for disease control and prevention
NHIS: National health insurance service
OR: Odds ratio
OSA: Obstructive sleep apnea
ROC: Receiver-operator characteristic
