# Supplementary material for: Impact of coronavirus disease-2019 on chronic respiratory disease in South Korea: an NHIS COVID-19 database cohort study
Source: BMC Pulm Med. 2021 Jan 6;21:12. doi: 10.1186/s12890-020-01387-1 (PMC7787421; doi:10.1186/s12890-020-01387-1)
Supplement: Supplementary file 1 — Additional File 1. The ICD-10 codes used by comorbidity to compute the Charlson comorbidity index [file 12890_2020_1387_MOESM1_ESM.docx]

**Additional File 1. The ICD-10 codes used by comorbidity to compute the Charlson comorbidity index**

The ICD-10 codes used by comorbidity to compute the Charlson comorbidity index are:

- Myocardial infarction: I21.x, I22.x, I25.2
- Congestive heart failure: I09.9, I11.0, I13.0, I13.2, I25.5, I42.0, I42.5 - I42.9, I43.x, I50.x, P29.0
- Peripheral vascular disease: I70.x, I71.x, I73.1, I73.8, I73.9, I77.1, I79.0, I79.2, K55.1, K55.8, K55.9, Z95.8, Z95.9
- Cerebrovascular disease: G45.x, G46.x, H34.0, I60.x - I69.x
- Dementia: F00.x - F03.x, F05.1, G30.x, G31.1
- Chronic pulmonary disease: I27.8, I27.9, J40.x - J47.x, J60.x - J67.x, J68.4, J70.1, J70.3
- Rheumatic disease: M05.x, M06.x, M31.5, M32.x - M34.x, M35.1, M35.3, M36.0
- Peptic ulcer disease: K25.x - K28.x
- Mild liver disease: B18.x, K70.0 - K70.3, K70.9, K71.3 - K71.5, K71.7, K73.x, K74.x, K76.0, K76.2 - K76.4, K76.8, K76.9, Z94.4
- Diabetes without chronic complication: E10.0, E10.1, E10.6, E10.8, E10.9, E11.0, E11.1, E11.6, E11.8, E11.9, E12.0, E12.1, E12.6, E12.8, E12.9, E13.0, E13.1, E13.6, E13.8, E13.9, E14.0, E14.1, E14.6, E14.8, E14.9
- Diabetes with chronic complication: E10.2 - E10.5, E10.7, E11.2 - E11.5, E11.7, E12.2 - E12.5, E12.7, E13.2 - E13.5, E13.7, E14.2 - E14.5, E14.7
- Hemiplegia or paraplegia: G04.1, G11.4, G80.1, G80.2, G81.x, G82.x, G83.0 - G83.4, G83.9
- Renal disease: I12.0, I13.1, N03.2 - N03.7, N05.2 - N05.7, N18.x, N19.x, N25.0, Z49.0 - Z49.2, Z94.0, Z99.2
- Any malignancy, including lymphoma and leukaemia, except malignant neoplasm of skin: C00.x - C26.x, C30.x - C34.x, C37.x - C41.x, C43.x, C45.x - C58.x, C60.x - C76.x, C81.x - C85.x, C88.x, C90.x - C97.x
- Moderate or severe liver disease: I85.0, I85.9, I86.4, I98.2, K70.4, K71.1, K72.1, K72.9, K76.5, K76.6, K76.7
- Metastatic solid tumour: C77.x - C80.x
- AIDS/HIV: B20.x - B22.x, B24.x
